# Determinants of antibiotic prescribing behaviors of primary care physicians in Hubei of China: a structural equation model based on the theory of planned behavior

**DOI:** 10.1186/s13756-019-0478-6

**Published:** 2019-01-30

**Authors:** Chenxi Liu, Chaojie Liu, Dan Wang, Zhaohua Deng, Yuqing Tang, Xinping Zhang

**Affiliations:** 1 School of Medicine and Health Management, Tongji Medical School, Huazhong University of Science and Technology, Wuhan, Hubei China; 2 School of Psychology and Public Health, La Trobe University, Melbourne, Vic Australia

**Keywords:** Primacy care, Antibiotic prescribing, Structural equation modelling, Theory of planned behaviors, Antibiotic resistance, China

## Abstract

**Background:**

Over-prescription of antibiotics is prevalent all over the world, contributing to the development of antibiotic resistance. The importance of understanding how physicians prescribe antibiotics is increasingly highlighted for the purpose of promoting good practice. This study aimed to identify factors that shape the antibiotic prescribing behaviors of physicians in primary care based on the theory of planned behavior (TPB).

**Methods:**

Data were collected from 503 prescribers within 65 primary care facilities in Hubei, tapping into four behavioral aspects leading to antibiotic prescribing based on TPB, namely, attitudes (the degree to which a prescriber is in favor of the use of antibiotics), subjective norms (perceived social pressure to which a prescriber is subject in relation to antibiotic prescriptions), perceived control of behaviors (how easy a prescriber feels in making a rational decision on antibiotic prescriptions) and intentions (the degree to which a prescriber is willing to prescribe antibiotics). A total of 440,268 prescriptions were audited to assess physician antibiotic prescribing practices. The four behavioral constructs were further linked with physician’s actual use of antibiotics using structural equation modelling (SEM) based on TPB.

**Results:**

On average, 40.54% (SD = 20.82%) of the outpatient encounters resulted in a prescription for an antibiotic given by the participants and 9.81% (SD = 10.18%) of the patients were given two or more antibiotics. The participants showing a more favorable attitude toward antibiotics had a higher intention to prescribe antibiotics (β = 0.226, *p* < 0.001) and a lower intention to reduce antibiotic use (β = − 0.211, *p* < 0.001). Those who perceived lower social pressure (β = 0.113, *p* = 0.030) and higher control over prescribing behaviors (β = 0.113, *p* = 0.037) reported a higher intention to reduce the use of antibiotics. However, such intention did not translate into prescribing practice (*p* > 0.05), although stronger perceived behavioral control was directly linked with lower antibiotic prescriptions (β = − 0.110, *p* = 0.019). Weaker perceived behavioral control was evident in the participants who showed a less favorable attitude toward antibiotics (β = 0.128, *p* = 0.001).

**Conclusion:**

Antibiotic prescribing practice is not under the volitional control of prescribers in primary care in China. Their rational prescribing practice is likely to be jeopardized by perceived weak control over prescribing behaviors.

**Electronic supplementary material:**

The online version of this article (10.1186/s13756-019-0478-6) contains supplementary material, which is available to authorized users.

## Background

Antibiotic products, commonly used in modern medical practices, have saved hundreds of millions of lives over the past century. However, the emergence of antibiotic resistant “super-bugs” in the recent few decades has resulted in increasing concerns over the arrival of a “post-antibiotic era” [1]. The lack of effective antibiotics will not only diminish our capacity to control infectious diseases, but also make many common medical procedures potentially fatal again [2]. It was estimated that over 700,000 people died in 2014 as a result of antibiotic resistance globally, and this figure could rise to 10 million by 2050, surpassing cancer as a leading cause of death [3].

The irrational and over-use of antibiotics in human beings and animals is blamed for fueling antibiotic resistance [4–6]. Over-prescription of antibiotics in medical services is widespread worldwide [7–9]. Some studies in the US revealed that up to 50% of antibiotic prescriptions in ambulatory settings can be deemed medically unnecessary [10, 11].

Researchers have attempted to understand how prescribing decisions are made using various behavioral theories, such as the knowledge-attitudes-practices (KAP) model, the operant learning theory (OLT), the social cognitive theory (SCT), the theory of reasoned action (TRA) and the theory of planned behavior (TPB). Godin and colleagues concluded in a systematic review that the TPB is the most appropriate model to predict prescribing behaviors [12]. Several studies applied the TPB to model the antibiotic prescribing decisions made by physicians [13–20]. However, many failed to link the model with actual prescription data [13, 18]. In addition, most studies were conducted in developed countries. Little is known about the antibiotic prescribing practice in developing countries, despite the fact that the misuse and overuse of antibiotics is more prevalent in these countries due to the poor competency of prescribers and low capacity of the regulation systems [21]. China is no exception. As a transitional economy and the world largest consumer of antibiotics, the widespread over-use of antibiotics, in particular in primary care, has become a serious issue of concern [22]. Each year, over 10 billion (50%) patient visits to primary care facilities involve antibiotic treatments [22, 23]. But less than 40% are appropriate [24].

This study aimed to establish a TPB model for antibiotic prescribing practice in primary care in Hubei of China. Such a model can provide evidence to support the design of interventional measures.

## Methods

### Settings

This study was conducted in Hubei, a province in central China with a population of 58.85 million. The social and economic development of Hubei ranks in the middle range of all the regions in China [23].

This study focused on the primary care sector, which attracts about 60% of all outpatient visits in China [23]. The study setting was restricted to urban community health centers (CHCs) and rural township health centers (THCs), two dominant forms of primary care facilities (most publicly owned). In Hubei, there are 342 CHCs and 1139 THCs, receiving 20.44 million and 58.00 million outpatient visits in 2016, respectively [23].

About 60% of outpatient visits to primary care facilities in Hubei involved an antibiotic prescription [25], which is higher than the national average of 50%. According to the World Health Organization [26], less than 30% of primary care visits would need an antibiotic prescription.

### Theoretical framework

**Fig. 1:**
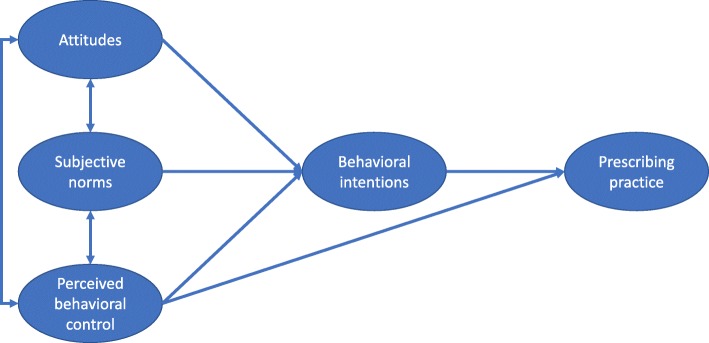
The TPB framework for antibiotic prescribing practice in primary care. Antibiotic prescribing practice is influenced by behavioral intentions and the perceived behavioral control of the prescribers based on the theory of planned behaviors, with the former serving as a motivational factor while the latter reflecting the ability of the prescribers to fulfill their intentions. Attitudes, subjective norms and perceived behavioral controls are linked to each other and they can influence the behavioral intentions of the prescribers

The theoretical framework was adapted from the TPB model (Fig. 1). The model included two indicators of prescribing practice: percentage of prescriptions containing antibiotics and percentage of prescriptions containing two or more antibiotics. The two indicators were proposed by the World Health Organization (WHO) for measuring the rational use of medicines [27]. Attitudes, subjective norms and perceived behavioral control in relation to antibiotic use were linked with intentions to prescribe antibiotics. They were deemed to be key factors shaping prescribing practice [28].

The TPB model assumed that antibiotic prescribing practice is influenced by behavioral intentions and the perceived behavioral control of the prescribers, with the former serving as a motivational factor while the latter reflecting the ability of the prescribers to fulfill their intentions. Attitudes, subjective norms and perceived behavioral controls are linked to each other and they can influence the behavioral intentions of the prescribers [28].

### Measurements

#### Dependent variables

Two indicators of prescribing practice were calculated by auditing the prescriptions in the participating facilities.

1. Percentage of prescriptions containing antibiotics = Number of prescriptions containing antibiotics/Number of total prescriptions × 100%
2. Percentage of prescriptions containing two or more antibiotics = Number of prescriptions containing two or more antibiotics/Total number of prescriptions × 100%

#### Independent variables: Constructs of TPB

A questionnaire was developed in line with the guidelines for TPB surveys [29]. It contained 23 question items (Additional file 1: Table S1), measuring attitudes, subjective norms, perceived behavioral control and behavioral intentions of the prescribers.

1. Attitudes (5 items)

Attitudes were defined as the degree to which a prescriber is in favor of the use of antibiotics in outpatient encounters [28]. Respondents were asked to rate on a five-point Likert scale the usefulness, appropriateness, responsiveness, harmfulness, and goodness of prescribing antibiotics for outpatients [14, 29]. A higher score indicates a more favorable attitude toward the use of antibiotics.

2. Subjective norms (6 items)

Subjective norms measure the perceived social pressure to which a prescriber is subject in relation to antibiotic prescriptions [28]. Respondents were asked to rate the degree of pressure arising from patients, colleagues and the society [14, 16, 29] on a five-point Likert scale, with a higher score indicating higher pressure.

3. Perceived behavioral control (5 items)

Perceived behavioral control measures how easy a prescriber feels in making a rational decision on antibiotic prescriptions [28]. It included self-efficacy, referring to the ability of a prescriber to prescribe antibiotics appropriately, and controllability, referring to the extent to which a prescriber can decide whether or not to prescribe antibiotics [14, 16, 30]. Respondents rated on a five-point Likert scale, with a higher score indicating higher behavioral control.

4. Behavioral intentions (6 items)

Behavioral intentions measure the degree to which a prescriber is willing to prescribe antibiotics [28]. Two subscales were developed asking whether a prescriber wants, expects, and plans to prescribe antibiotics and to reduce antibiotic prescriptions, respectively [29]. Respondents rated on a five-point Likert scale, ranging from “strongly disagree” (1) to “strongly agree” (5).

The questionnaire was piloted on 21 physicians from three primary care facilities. The participants were asked to complete the questionnaire and provide feedback on the clarity and difficulty of items, which resulted in some revisions to the items. The reliability and validity of the revised questionnaire were tested in the final survey (*n* = 503). The confirmatory factor analysis (CFA) demonstrated an excellent fitness of data into the proposed model: root mean square error of approximation (RMSEA) = 0.039 (< 0.05); Tucker–Lewis index (TLI) = 0.993 (> 0.95); comparative fit index (CFI) = 0.991 (> 0.95) and weighted root mean square residual (WRMR) = 0.934 (< 1) [31, 32]. High internal consistency was indicated by the high Cronbach’s alpha (0.792–0.913) for the measured constructs.

### Participants

All physicians from the sampled primary care facilities were invited to participate in the survey. Those who met the following criteria were included in this study:

1. being able to prescribe antibiotics independently;
2. Having authorized ≥100 prescriptions over the past three months prior to the survey (to ensure a reliable estimation)

### Sample size and sampling

A multi-stage cluster sampling strategy was adopted. The first stage involved a random selection of one urban city and two rural counties in western, central and eastern Hubei, respectively.

We estimated that a minimal sample size of 6 primary care facilities in each city/county involving 23 respondents was required according to the calculator developed by Dhand and Khatkar (with an expected deviation < 4, precision = 1, level of confidence = 95%, inter-class correlation coefficient < 0.02 and cluster size = 4) [33]. We increased the sample size to 70 respondents per city/county considering that only some (about 40% according to a pilot study) of the physicians would meet the inclusion criteria. As a result, the participating primary care facilities in each city/county increased from six to eight. They were randomly selected in the second stage. All primary care facilities were included if a city/county had less than eight primary care facilities.

In the third stage, trained interviewers were paired and dispatched to the selected CHCs and THCs. All of the physicians on duty were approached and invited to participate in the survey. If less than 70 respondents were found in a city or county from the selected primary care facilities, an additional primary care facility (if available) was added to the sample. Eventually, 19 urban CHCs and 46 rural THCs were involved in this study.

### Data collection

Prescription data of the participating primary care institutions from January 1st to March 31st, 2018 (three months prior to the survey of the prescribers) were extracted from the local governments and the participating institutions. We calculated the two prescribing indicators for each prescriber.The approached physicians who prescribed medicines were asked to complete the questionnaire independently. Written informed consent was obtained by the interviewers from each respondent prior to the survey. On average, the survey took around 10 min to complete. The returned questionnaires were examined by the interviewers to ensure completeness before a token gift (roughly $1.65) was given to the participant.

In total, 712 questionnaires were distributed and 664 were returned. After matching with the prescription data, 503 met the inclusion criteria and were included for final analyses. This resulted in an effective response rate of 71%.

### Data analysis

We calculated the mean score and standard deviation for each measurement. A mean score of 3 represented a neutral response (Additional file 2: Table S2).

**Fig. 2:**
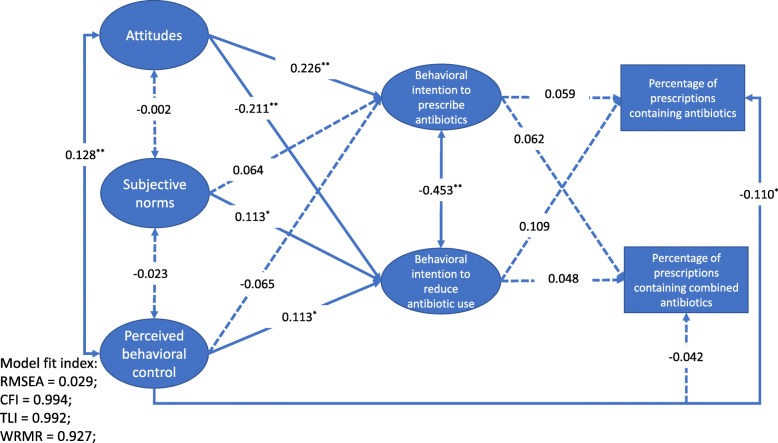
Structure equation model on antibiotic prescribing practice based on the theory of planned behavior. Standardized path coefficients were presented; the solid lines indicate the paths with statistical significance; *p < 0.05; **p < 0.01

Structural equation modelling (SEM) analyses were performed based on the proposed TPB model (Fig. 2). Means and variance adjusted weighted least squares (WLSMV) estimation were adopted, which was designed for ordinal data (e.g. five-point Likert scale) [34]. We used a mixed model, adjusting for the cluster effect (at the facility level). The fitness of data into the SEM model was assessed using several recommended criteria [31, 32]: RMSEA< 0.05; TLI > 0.95; CFI > 0.95 and WRMR< 1.

The statistical analyses were performed using STATA (version 12.0) and Mplus (version 6.0). A *p* value < 0.05 was considered statistically significant.

## Results

### Characteristics of respondents

The respondents had a mean age of 43.40 years, with a standard deviation (SD) of 9.57. Most of the respondents were male (70.58%) and worked in THCs (78.53%). On average, the respondents had engaged in clinical practice for 16.28 (SD = 10.08) years. Only a small proportion (10.14%) of the respondents held a senior title (equivalent to a professorial title). The majority (74.95%) had received some training in regard to the use of antibiotics in the year prior to the survey (Table 1).

**Table 1:** Characteristics of respondents

Characteristics	
Age, years (Mean ± SD*)	43.40 ± 9.57
Gender, N(%)	
Male	355 (70.58)
Female	148 (29.42)
Setting, N(%)	
Community health center	108 (21.47)
Township health center	395 (78.53)
Job title, N(%)	
Doctor	257 (51.09)
Attending doctor	195 (38.77)
Associate chief or chief doctor	51 (10.14)
Education, N(%)	
High school and below	43 (8.55)
Associate degree or diploma	267 (53.08)
University degree	193 (38.37)
Annual household income, N(%)	
¥ < 40,000	144 (28.63)
¥ 40,000–79,999	255 (50.70)
¥ 80,000-119,999	78 (15.51)
¥ ≥120,000	26 (5.17)
Years of clinical practice (Mean ± SD*)	16.28 ± 10.08
Training regarding antibiotics over last year, N(%)	
Yes	377 (74.95)
No/Don’t know	126 (25.05)

^*^SD Standard Deviation

### Prescribing practices and measurement scores of respondents

A total of 440,268 prescriptions were audited, with 194,983 containing an antibiotic and 40,919 containing two or more antibiotics, respectively. On average, the eligible prescribers authorized 875 (ranging from 100 to 7145) prescriptions each. About 40.54% (SD = 20.82%) of the prescriptions contained an antibiotic and 9.81% (SD = 10.18%) contained two or more antibiotics (Table 2).

**Table 2:** Prescribing practices and measurement scores of respondents

Measurements	Mean (SD)	Skewness	Median	N (%) of scores > 3
Attitudes (5 items)	3.14 (0.79)	−0.2963	3.20	256 (50.89)
Subjective norms (6 items)	3.05 (0.70)	−0.2584	3.00	245 (48.71)
Perceived behavioral control (5 items)	3.39 (0.67)	−0.1065	3.40	336 (66.80)
Behavioral intentions				
to prescribe antibiotics (3 items)	2.15 (0.63)	0.2699	2.00	21 (4.17)
to reduce antibiotic prescriptions (3 items)	4.29 (0.52)	−0.1684	4.00	487 (96.82)
Prescribing practice				
Percentage of prescriptions containing antibiotics	40.54 (20.82)	0.3171	40.35	–
Percentage of prescriptions containing combined antibiotics	9.81 (10.18)	2.3259	7.20	–

*SD* Standard deviation

The attitudes of the respondents showed a slight inclination in favor of the use of antibiotics (Mean = 3.14, SD = 0.79). Their perceptions of social pressure (subjective norms) on antibiotic prescriptions were almost neutral (Mean = 3.05, SD = 0.70). The majority (66.8%) felt positive in behavioral control (Mean = 3.39, SD = 0.67).

A low intention to prescribe antibiotics (Mean = 2.15, SD = 0.63) and high intentions to reduce antibiotic prescriptions (Mean = 4.29, SD = 0.52) were evident.

### Structural equation modelling

A very good fitness of data into the TPB model was found: RMSEA = 0.029 (< 0.05); CFI = 0.994 (> 0.95); TLI = 0.992 (> 0.95) and WRMR = 0.927 (< 1).

The TPB model (Fig. 2) indicated that an attitude in favor of antibiotics was linked to higher intentions to prescribe antibiotics (β = 0.226, *p* < 0.001) and lower intentions to reduce antibiotic prescriptions (β = − 0.211, *p* < 0.001). Greater social pressures (subjective norms) were linked to higher intentions to reduce antibiotic prescriptions (β = 0.113, *p* = 0.030). A greater sense of control over behaviors was associated with higher intentions to reduce antibiotic prescriptions (β = 0.113, *p* = 0.037) and a lower percentage of prescriptions containing antibiotics (β = − 0.110, *p* = 0.019). However, intentions did not show significant associations with the two indicators for prescribing practice (*p* > 0.05).

## Discussion

### Main findings

This study demonstrated that physicians over-prescribed antibiotics in primary care settings in Hubei of China, with 40% of prescriptions containing an antibiotic and 10% containing two and more antibiotics, respectively. These rates are much higher than the recommended levels proposed by the WHO [25]. The combined use of antibiotics should be minimal given that patients visiting primary care facilities usually have a minor or common illness [35].

The TPB model fits well with the survey data: intentions to prescribe antibiotics are predicted by the attitudes, subjective norms and sense of behavioral control of the prescribers. However, such intentions are not directly linked to actual prescribing behaviors (*p* > 0.05). The reduction of actual use of antibiotics is shaped by higher levels of behavioral control, a feeling of being able to translate intentions into practices (β = − 0.110, *p* = 0.019).

### Comparison with other studies

Several studies have used the TPB model to explore physician behaviors relevant to antibiotic use, including antibiotic prescribing, choice of appropriate antibiotics and adherence to guidelines for antibiotic usage. Two of these studies established a TPB model predicting actual prescribing behaviors. The study conducted in the US concluded that intentions play a limited role in the choice of antibiotics by general practitioners [36]. The other study from Scotland found out that a high level of behavioral intentions combined with a low level of behavioral control drive the (irrational) antibiotic prescribing for patients with upper respiratory tract infections (URTIs) [13].

It is important to note that physicians working in different health systems are subject to different working environments and social pressures. Our study in the Chinese setting provides additional evidence to illustrate the limited role of intentions and the importance of perceived behavioral control in shaping prescribing decisions. Our study drew conclusions based on a large dataset, involving a vigorous validation process of measurements.

### Interpretation and policy implications

#### Limited effects of behavioral intentions

It appears that antibiotic prescribing practice is not under the volitional control of primary care physicians in Hubei of China. Interpretation of the lack of a direct link between intentions and practices in antibiotic prescribing needs to be cautious. In this study, we did not distinguish between appropriate and inappropriate antibiotic prescriptions. This may lead to underestimation of the association between behavioral intentions and irrational prescribing practices for antibiotics, because rational use of antibiotics is driven by clinical needs of the patients, not intentions of the physicians [37]. It is also likely that a stronger link between intentions and prescribing practices for antibiotics may exist for certain disease conditions than for others. A previous study revealed that disease-specific prescribing guidelines such as those for URTIs can facilitate the translation of intentions into practices [13]. However, intentions play a much less significant role if the physicians are not able to make a clear diagnosis and the over-prescription of antibiotics is indeed linked with the clinical capacity of the physicians (measured by behavioral control). Similar findings were also reported in a study conducted in the US [36].

Empirical evidence shows that prescribing decisions are likely to be a result of interactive effects among knowledge, skills, communications and policy restrictions [37, 38]. Antibiotic prescribing considers not only intrinsic factors from the physicians but also external factors from the patients and the healthcare system [37, 38]. Paula recommended a more comprehensive behavioral model involving constructs from the perspectives of physicians, patients, healthcare systems, and pharmaceutical industries [37]. But it is challenging to test such as model due to difficulties in data collection.

#### Effects of perceived behavioral control

The TPB model established in this study shows that perceived behavioral control is the only significant pathway that influences prescribing practice: a higher sense of control reduces antibiotic prescriptions. Perceived behavioral control reflects the self-efficacy and controllability of physicians on prescribing behaviors [30]. Physicians may have varied understanding about rational use of antibiotics. Such varied understanding would inevitably influence their prescribing decisions [39]. Many environmental factors, such as preferred drug list, financial incentives, and patient demands, also can influence physician’s controllability of antibiotic prescribing, resulting in different prescribing decisions as well [21, 36, 40, 41].

Overall, participants in this study reported a high level of perceived behavioral control. However, one-third of them felt uneasy to prescribe antibiotics in line with their clinical knowledge. The Chinese government imposed strong restrictive measures on antibiotic prescribing through its zero-mark-up policy for sales of medicines [42] and practice guidelines [43–45]. But limited attention has been attached to the management of patient demands and expectations. A systematic review confirmed that patient demands are a significant contributor to over-prescription of antibiotics [37]. Delayed antibiotic prescribing strategies have attracted increasing attention in recent years as a compromised measure for managing consumer demands [46, 47]. Further studies on the effectiveness of such strategies in China are warranted given that the lack of trust and intense patient-doctor relationships have become a major concern in China [48].

### Strengths and limitations

This study contained a large representative sample of physicians and their prescriptions, which is essential to ensure a reliable estimate of prescribing behaviors.

There are several limitations in this study. First, this study was conducted in primary care facilities in Hubei of China. Regional disparities may prevent us from extrapolating the findings to other population. Secondly, we did not assess appropriateness of prescriptions due to the lack of diagnostic information. We were not able to measure Defined Daily Dose of prescribed medicines either. Finally, we did not explore determinants of the four constructs measured in the TPB model. Further studies are warranted.

## Conclusion

Over-prescription of antibiotics is evident in primary care settings in Hubei of China, in particular in relation to the combined use of antibiotics. However, the antibiotic prescribing practice is not under the volitional control of the prescribers. Their rational prescribing practice is likely to be jeopardized by perceived weak control over prescribing behaviors. Policy interventions should give equal considerations on prescribing capacity building and the management of pressure arising from perverse (e.g. financial) incentives and consumer demands.

## Additional files

Additional file 1: **Table S1.** Survery instruments (Translated version). (DOCX 29 kb)

Additional file 2: Detailed information of physicians’ responses to items based on TPB. (DOCX 19 kb)

## Abbreviations

CFA: Confirmatory factor analysis
CFI: Comparative fit index
CHCs: Community health centers
KAP: Knowledge-attitudes-practices
OLT: Operant learning theory
RMSEA: Root mean square error of approximation
SCT: Social cognitive theory
SD: Standard deviation
SEM: Structural equation modelling
THCs: Township health centers
TLI: Tucker–Lewis index
TPB: Theory of planned behavior
TRA: Theory of reasoned action
URTIs: Upper respiratory tract infections
WHO: World Health Organization
WLSMV: Means and variance adjusted weighted least squares
WRMR: Weighted root mean square residua
