# Supplementary material for: Determinants of antibiotic prescribing behaviors of primary care physicians in Hubei of China: a structural equation model based on the theory of planned behavior
Source: Antimicrob Resist Infect Control. 2019 Jan 30;8:23. doi: 10.1186/s13756-019-0478-6 (PMC6354420; doi:10.1186/s13756-019-0478-6)
Supplement: Supplementary file 1 — Table S1. Survery instruments (Translated version). (DOCX 29 kb) [file 13756_2019_478_MOESM1_ESM.docx]

**Survey of physician’s behavior towards antibiotic prescribing in primary cares**

**Dear physicians：**

Greeting!

We are conducting a survey to understand physician’s behavior towards antibiotic prescribing. We invited you to complete this 10-minute questionnaire and your responses are of great values. Your personal information would be confidential and the responses were only used for academic research only. Thanks very much for your participation. Please note that **the following questions do not have the correct answer**, please fill in them according to your personal situation / attitude.

School of Medicine and Health Management

Tongji Medical School, Wuhan

**Part 1: Attitudes**

| 1. I think prescribing outpatients antibiotics is | | | | |
| --- | --- | --- | --- | --- |
| 1.1 □ Very useful | □ Useful | □ Neutral | □ Useless | □ Very useless |
| 1.2 □ Very appropriate | □ Appropriate | □ Neutral | □ Inappropriate | □ Very inappropriate |
| 1.3 □ Very irresponsible | □ Irresponsible | □ Neutral | □ Responsible | □ Very responsible |
| 1.4 □ Very harmful | □ Harmful | □ Neutral | □ Beneficial | □ Very beneficial |
| 1.5 □ Very bad | □ Bad | □ Neutral | □ Good | □ Very good |

**Part 2: Subjective norms**

| - 1. Outpatients want me to prescribe antibiotics to them | | | | |
| --- | --- | --- | --- | --- |
| □ Always | □ Often | □ Sometimes | □ Rarely | □ Never |
| - 1. Outpatients think I should prescribe antibiotics to them | | | | |
| □ Always | □ Often | □ Sometimes | □ Rarely | □ Never |
| - 1. My colleagues prescribe antibiotics to outpatients | | | | |
| □ Always | □ Often | □ Sometimes | □ Rarely | □ Never |
| - 1. My colleagues ____ think I should prescribe antibiotics to outpatients | | | | |
| □ Always | □ Often | □ Sometimes | □ Rarely | □ Never |
| - 1. It is ____ expected of me that I should prescribe antibiotics to outpatients | | | | |
| □ Always | □ Often | □ Sometimes | □ Rarely | □ Never |
| - 1. I feel under social pressure to prescribe antibiotics for outpatients | | | | |
| □ Always | □ Often | □ Sometimes | □ Rarely | □ Never |

**Part 3: Perceived behavioral control**

| - 1. How much control that I have when decide prescribe antibiotics to outpatients or not? | | | | |
| --- | --- | --- | --- | --- |
| □ Full control | **□** Most control | **□** Some control | **□** Little control | **□** No control |
| - 1. Whether prescribe antibiotics to outpatients or not is entirely up to me? | | | | |
| □ Strongly agree | **□** Agree | **□** Neutral | **□** Disagree | **□** Strongly disagree |
| - 1. Decide whether prescribe outpatients antibiotics was ___ to me | | | | |
| □ Very easy | **□** Easy | **□** Neutral | **□** Difficult | **□** Very difficult |
| - 1. I feel capable to decide whether prescribe outpatients antibiotics or not | | | | |
| □ Strongly agree | **□** Agree | **□** Neutral | **□** Disagree | **□** Strongly disagree |
| - 1. It is easy for me to decide whether prescribe outpatients antibiotics or not | | | | |
| □ Strongly agree | **□** Agree | **□** Neutral | **□** Disagree | **□** Strongly disagree |

**Part 4: Behavioral intentions**

|  | Very agree | Agree | Neutral | Disagree | Very Disagree |
| --- | --- | --- | --- | --- | --- |
| - 1. I want to reduce antibiotic use for outpatients | □ | □ | □ | □ | □ |
| - 1. I expect to reduce antibiotic use for outpatients | □ | □ | □ | □ | □ |
| - 1. I plan to reduce antibiotic use for outpatients | □ | □ | □ | □ | □ |
| - 1. I want to prescribe antibiotic to outpatients | □ | □ | □ | □ | □ |
| - 1. I expect to prescribe antibiotic to outpatients | □ | □ | □ | □ | □ |
| - 1. I plan to prescribe antibiotic to outpatients | □ | □ | □ | □ | □ |
