# Supplementary material for: Determinants of antibiotic prescribing behaviors of primary care physicians in Hubei of China: a structural equation model based on the theory of planned behavior
Source: Antimicrob Resist Infect Control. 2019 Jan 30;8:23. doi: 10.1186/s13756-019-0478-6 (PMC6354420; doi:10.1186/s13756-019-0478-6)
Supplement: Supplementary file 2 — Detailed information of physicians’ responses to items based on TPB. (DOCX 19 kb) [file 13756_2019_478_MOESM2_ESM.docx]

| **Table S2 Detailed information of physicians’ responses to items based on TPB** | | | | | |
| --- | --- | --- | --- | --- | --- |
| **Measured characteristics and corresponding items** | **Responses (N, %)** | | | | |
| **1. Attitudes (I think prescribing outpatients antibiotics is)** | **Very positive** | **Positive** | **Neutral** | **Negative** | **Very negative** |
| 1.1 Very useful – Very useless | 28 (5.53) | 211 (41.70) | 191 (37.75) | 66 (13.04) | 10 (1.98) |
| 1.2 Very appropriate – Very inappropriate | 26 (5.14) | 191 (37.75) | 165 (32.61) | 110 (21.74) | 14 (2.77) |
| 1.3 Very responsible – Very irresponsible | 32 (6.32) | 180 (35.57) | 174 (34.39) | 92 (18.18) | 28 (5.53) |
| 1.4 Very beneficial – Very harmful | 12 (2.37) | 145 (28.66) | 215 (42.49) | 107 (21.15) | 27 (5.34) |
| 1.5 Very good – Very bad | 6 (1.19) | 132 (26.09) | 198 (39.13) | 132 (26.09) | 38 (7.51) |
| **2. Subjective norms** | **Always** | **Often** | **Sometimes** | **Rarely** | **Never** |
| 2.1 Outpatients want me to prescribe antibiotics to them | 73 (14.43) | 243 (48.02) | 140 (27.67) | 41 (8.10) | 9 (1.78) |
| 2.2 Outpatients think I should prescribe antibiotics to them | 63 (12.45) | 155 (30.63) | 203 (40.12) | 71 (14.03) | 14 (2.77) |
| 2.3 My colleagues prescribe antibiotics to outpatients | 11 (2.17) | 124 (24.51) | 282 (55.73) | 82 (16.21) | 7 (1.38) |
| 2.4 My colleagues ____ think I should prescribe antibiotics to outpatients | 10 (1.98) | 59 (11.66) | 241 (47.63) | 147 (29.05) | 49 (9.68) |
| 2.5 It is ____ expected of me that I should prescribe antibiotics to outpatients | 27 (5.34) | 129 (25.49) | 139 (27.47) | 122 (24.11) | 89 (17.59) |
| 2.6 I feel under social pressure to prescribe antibiotics for outpatients | 35 (6.92) | 116 (22.92) | 139 (27.47) | 106 (20.95) | 110 (21.74) |
| **3. Perceived behavioral control** | **Very positive** | **Positive** | **Neutral** | **Negative** | **Very negative** |
| 3.1 How much control that I have when decide prescribe antibiotics to outpatients or not? | 96 (18.97) | 249 (49.21) | 109 (21.54) | 47 (9.29) | 5 (0.99) |
| 3.2 Whether prescribe antibiotics to outpatients or not is entirely up to me? | 42 (8.30) | 183 (36.17) | 135 (26.68) | 122 (24.11) | 24 (4.74) |
| 3.3 Decide whether prescribe outpatients antibiotics was ___ to me | 29 (5.73) | 152 (30.04) | 230 (45.45) | 90 (17.79) | 5 (0.99) |
| 3.4 I feel capable to decide whether prescribe outpatients antibiotics or not | 43 (8.50) | 313 (61.86) | 99 (19.57) | 50 (9.88) | 1 (0.20) |
| 3.5 It is easy for me to decide whether prescribe outpatients antibiotics or not | 21 (4.15) | 181 (35.77) | 143 (28.26) | 149 (29.45) | 12 (2.37) |
| **4. Behavioral intentions** | **Very agree** | **Agree** | **Neutral** | **Disagree** | **Very disagree** |
| 4.1 I want to reduce antibiotic use for outpatients | 194 (38.34) | 292 (57.71) | 17 (3.36) | 3 (0.59) | 0 (0.00) |
| 4.2 I expect to reduce antibiotic use for outpatients | 196 (38.74) | 289 (57.11) | 20 (3.95) | 1 (0.20) | 0 (0.00) |
| 4.3 I plan to reduce antibiotic use for outpatients | 147 (29.05) | 309 (61.07) | 48 (9.49) | 2 (0.40) | 0 (0.00) |
| 4.4 I want to prescribe antibiotic to outpatients | 0 (0.00) | 14 (2.77) | 126 (24.90) | 309 (61.07) | 57 (11.26) |
| 4.5 I expect to prescribe antibiotic to outpatients | 0 (0.00) | 13 (2.57) | 98 (19.37) | 314 (62.06) | 81 (16.01) |
| 4.6 I plan to prescribe antibiotic to outpatients | 0 (0.00) | 16 (3.16) | 134 (26.48) | 274 (54.15) | 82 (16.21) |
